# Al-Awadi/Raas-Rothschild Syndrome in a Newborn with Additional Anomalies

**DOI:** 10.4274/jcrpe.v2i1.49

**Published:** 2010-12-08

**Authors:** Esma Alp, Hayrullah Alp, Mehmet Emre Atabek, Özgür Pirgon

**Affiliations:** 1 Selçuk University Meram Medical Faculty, Department of Pediatrics, Konya, Turkey; 2 Selçuk University Meram Medical Faculty, Department of Pediatric Endocrinology, Konya, Turkey; 3 Konya Research and Training Hospital, Department of Pediatric Endocrinology, Konya, Turkey; +90 332 223 63 10+90 332 223 61 82drhayrullahalp@hotmail.comSelçuk University Meram Medical Faculty, Department of Pediatrics, Konya, Turkey

**Keywords:** Al-Awadi/Raas-Rothschild syndrome, phocomelia, pelvic agenesis

## Abstract

Al-Awadi/Raas-Rothschild (AARR) syndrome is a rare phocomelia syndrome characterized by limb/pelvic hypoplasia/aplasia, renal anomalies such as horseshoe and polycystic kidney, and abnormal facial features including cleft palate, hypertelorism and micro-retrognatia. Autosomal recessive inheritance has been proposed for AARR syndrome. In this report a boy affected with AARR syndrome is presented. The previous pregnancy of the mother was terminated because of lower limb agenesis detected at 14th week of gestation. This report emphasizes the importance of recognizing severe pelvic and limb deficiencies in newborns with AARR syndrome and differentiating the syndrome from other multiple malformation syndromes. Fetal ultrasonography at 15th week of gestation is helpful in diagnosing the major extremity anomalies in the fetus.

**Conflict of interest:**None declared.

## INTRODUCTION

Al-Awadi/Raas-Rothschild (AARR) syndrome is a distinct multiple malformation syndrome that includes severe defects of the limbs and pelvis, craniofacial anomalies and renal or uterine malformations. It was reported by Al-Awadi at al (1) in 1985 and Raas-Rothschild et al (2) in 1988. Severe limb deficiencies have been reported in other well-known genetic entities such as the Roberts/SC phocomelia syndrome, Zimmer phocomelia, Schinzel phocomelia, Baller-Gerold syndrome, X-linked amelia, thalidomide embryopathy (3). All these multiple malformation syndromes can be distinguished from one another by clinical and radiological evaluation. 

The aim of the present report of a patient with AARR syndrome is to emphasize the distinctiveness of the limb and pelvic defects encountered in this syndrome from those seen in other rare multiple malformation syndromes associated with intercalary limb deficiencies. This report also illustrates that an absent pelvis and lower limbs seen on the ultrasound of a fetus may suggest this syndrome prenatally.

## PATIENT REPORT

The affected case was a Turkish boy born to nonconsanguineous parents. He was the second living child of the family born from the third pregnancy of the mother. The mother’s first child was a girl and she was healthy, but afetus with lower limb agenesis was diagnosed during the second pregnancy and the pregnancy was terminated. 

However, autopsy or radiological evaluation was not made in that fetus. There was no other family history of skeletal defects, mental retardation and recurrent pregnancy losses. The mother took only vitamins during her pregnancy and denied use of other medications or exposure to known teratogenic agents. She had no history of chronic illnesses.

Sonographic screening of our case in utero at 15^th^ week of pregnancy revealed bilateral absence of lower limbs. The family did not give consent to termination of the pregnancy. The patient was born at 38 weeks of gestation by caesarean section. Birth weight was 2060 g (<3^rd^ percentile), head circumference was 34.5 cm (50-75^th^ percentile) and crown-to-rump length was 31 cm. (<3^rd^ percentile). The lower limbs and pelvic structures were not palpable. The upper limb structures were totally normal on examination (Figure 1a). The X-rays showed that the pelvis and femurs were not present, while the bones of both upper limbs and vertebral column were normal (Figure 1b). Craniofacial anomalies included incomplete cleft palate, hypertelorism and micro-retrognatia. Other examination findings were cryptorchidism (testis were not palpated in the scrotum) and macrophallus (penile length was 4 cm). Ultrasonography of the abdomen revealed horseshoe kidneys. The testicles were in the inguinal canal. Magnetic resonance imaging findings of the brain and pituitary gland were normal. Karyotype was 46, XY. The infant was followed in the newborn intensive care unit and died from aspiration pneumonia on the 5^th^ day of life.

**Figure 1a:**
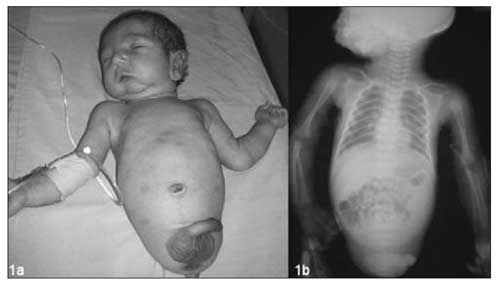
The general appearance of the patient (absent lower limbs, normal upper limbs, macrophallus and craniofacial findings)

**Figure 1b:**
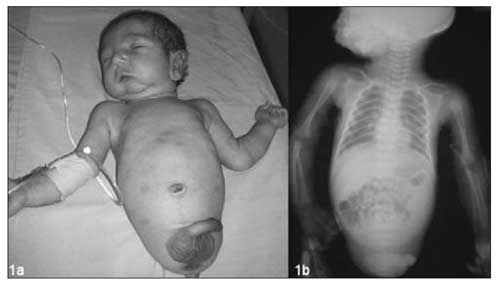
Radiogram showing agenesis of the pelvic bone and of the bones of lower limbs

## DISCUSSION

The clinical findings in our case confirm the diagnosis of AARR syndrome. Additional anomalies encountered in our patient, such as retro-migrognatia, incomplete cleft palate, hyperteleorism, cryptorchidism, macrophallus and horseshoe kidney, are rare in this syndrome (1, 2). The history of the second and the last pregnancies of the mother did not reveal exposure to specific teratogenic agents such as thalidomide. The clinical findings were also not consistent with thalidomide embryopathy.

The prevalence of bilateral lower limb aplasia is 0.4/100 000 (4). Cases with limb/pelvis hypoplasia/aplasia were first reported by Al-Awadi et al (1). In 1988 Raas-Rothschild et al (2) described the same malformations in some other families. The clinical features of this multiple malformation syndrome are pelvic aplasia or hypoplasia, intercalary limb deficiencies, craniofacial anomalies and genitourinary defects. The phenotypic findings of our patient led us to the diagnosis of a new case of AARR syndrome. 

The distinctive features of other multiple malformation syndromes should be distinguished from AARR syndrome (Table 1). Zimmer phocomelia includes severe craniofacial anomalies, imperforate anus, genital abnormalities and pelvic hypoplasia, and was first reported in 1985 (5). Major cranial malformations in this syndrome are anencephaly, meningoencephalocele and complete absence of frontal bones. Cleft palate, severe ear and nasal hypoplasia are the additive clinical findings. Tetraphocomelia in Zimmer phocomelia syndrome was described by Kosaki et al (6). Schinzel phocomelia is characterized by limb/pelvis hypoplasia/aplasia, intercalary limb deficiencies and large parietooccipital skull defects without meningocele, encephalocele or other brain malformations (7). Cryptorchidism, renal anomaly and miscellaneous abnormal facial features are not usual in Schinzel phocomelia. Oral cleft was reported in only one patient. Roberts/SC phocomelia includes oral clefts, ear malformations, microcephaly, cryptorchidism and characteristic ‘puffing’ of heterochromatic regions near centromeres seen in the chromosomal analyses of most affected individuals (8). 

Autosomal recessive inheritance has been proposed for AARR syndrome, Roberts/SC phocomelia, Schinzel phocomelia and Zimmer phocomelia. Consanguinity has been documented in most cases with AARR syndrome (1, 2). Also some families with Schinzel phocomelia syndrome had two affected siblings born to healthy parents (3, 7, 9). Woods et al (10) reported homozygous missense mutations in the dorsoventral-patterning gene WNT7A in Fuhrmann, Al-Awadi/Raas-Rothschild and Schinzel phocomelia syndromes. The family history of the patient with AARR syndrome presented in this report revealed that the mother’s second pregnancy was terminated due to detection of the lower limb agenesis in the fetus. Eventually, this documentation verifies the autosomal recessive inheritance. The hypoplasia/aplasia of the pelvis and limbs seen in fetuses on ultrasonography performed by an experienced physicians at 15^th^ week of pregnancy will provide helpful clues for prenatal detection of major extremity anomalies including AARR syndrome.

**Table 1:**
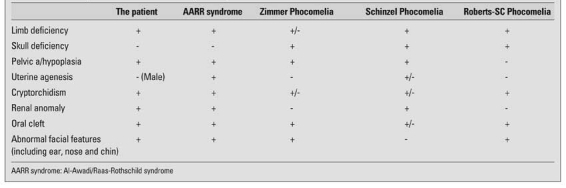
The clinical patterns of malformations in specific syndromes
